# Anticipating transitions in mental health in at-risk youth: A large-scale diary study into early warning signals

**DOI:** 10.1192/j.eurpsy.2021.1215

**Published:** 2021-08-13

**Authors:** M. Schreuder, J. Wigman, A. Smit, C. Hartman, M. Wichers

**Affiliations:** Department Of Psychiatry, Interdisciplinary Center Psychopathology And Emotion Regulation (icpe), University of Groningen, University Medical Center Groningen, Groningen, Netherlands

**Keywords:** diary study, complex systems, transdiagnostic psychopathology, early warning signals

## Abstract

**Introduction:**

Transitions in mental health, such as the onset or sudden progression of psychopathology, are difficult to foresee. If mental health behaves like other complex systems, drops in mental health may be anticipated by early warning signals (EWS), which manifest in the dynamics of time series data.

**Objectives:**

This study aimed to establish the sensitivity and specificity of EWS as personalized risk markers for sudden drops mental health.

**Methods:**

Individuals (N=122, mean age 23.6 ±0.7 years, 57% males) at increased risk for psychopathology completed daily questionnaires on mental states for six consecutive months. Transitions in mental health were identified by change point analyses. EWS, operationalized as rising trends in the autoregressive coefficient of 36 negative mental states, were identified using generalized additive models.

**Results:**

EWS were found for 59% of individuals with a drop in mental health, and for 47% without such a drop (sensitivity: 0-.12; specificity: .88-1). There were considerable individual differences in the prevalence, strength, and timing of EWS.

**Conclusions:**

EWS might be informative of impeding transitions, yet they are also highly conservative. Present findings may inspire future research into the prerequisites for detecting EWS in the context of mental health, for instance with respect to the stability of pre- and post-transition phases, the magnitude of transitions, and the timescale at which EWS manifest. An improved understanding of the dynamics that govern psychopathology could ultimately allow us to determine whether a specific individual at a specific moment in time is at risk for a sudden onset or progression of mental health problems.

